# Supplementary figures and images for: A comparison of aquaporin expression in mosquito larvae (Aedes aegypti) that develop in hypo-osmotic freshwater and iso-osmotic brackish water
Source: PLoS One. 2020 Aug 20;15(8):e0234892. doi: 10.1371/journal.pone.0234892 (PMC7440623; doi:10.1371/journal.pone.0234892)

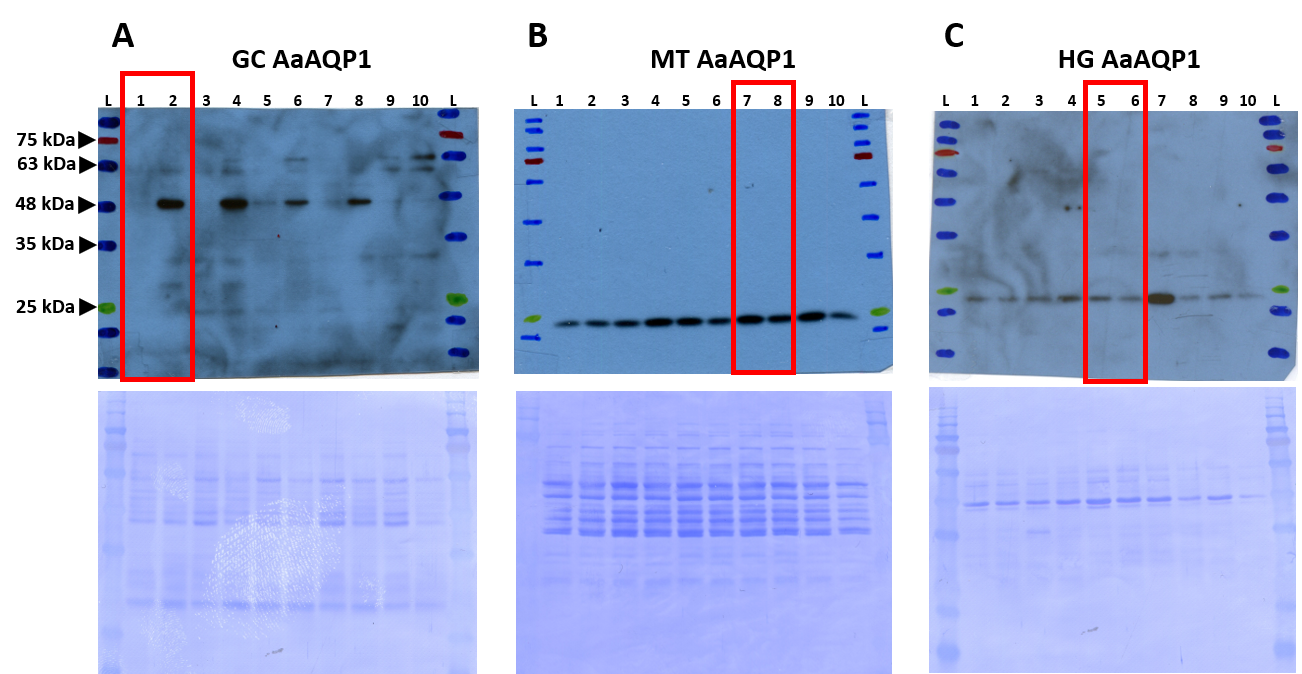

Supplement: S1 Fig — Five biological replicates of organ protein homogenates from freshwater reared larvae are loaded in lanes 1, 3, 5, 7 and 9. Five biological replicates of organ protein homogenates from brackish water reared larvae are loaded in lanes 2, 4, 6, 8 and 10. The red rectangle indicates the lanes shown in Fig 2. For protein quantification of AaAQP1 the ~ 23 kDa band was used for MT and HG and the ~50 kDa band was used for GC. L = Ladder; GC = gastric caeca; MT = Malpighian tubules; HG = Hindgut. (TIF) [file pone.0234892.s001.tif]

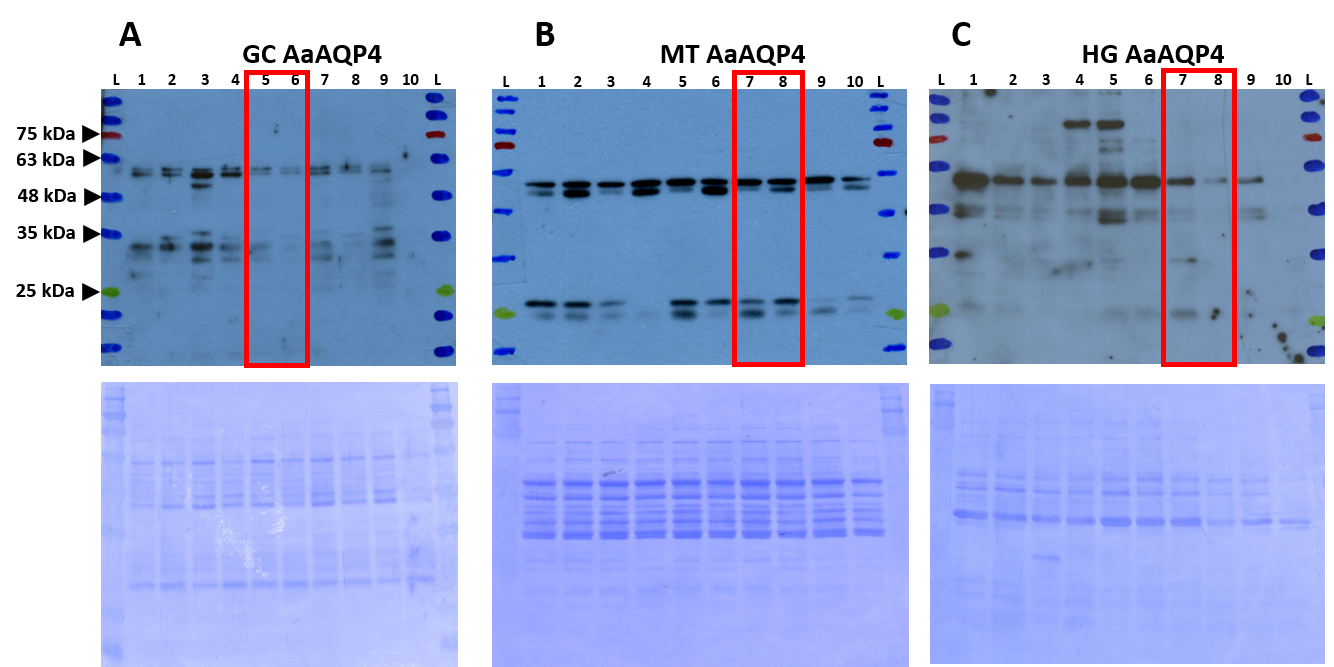

Supplement: S2 Fig — Five biological replicates of organ protein homogenates from freshwater reared larvae are loaded in lanes 1, 3, 5, 7 and 9. Five biological replicates of organ protein homogenates from brackish water reared larvae are loaded in lanes 2, 4, 6, 8 and 10. The red rectangle indicates the lanes shown in Fig 3. For quantification of AaAQP4 protein the band intensities of the putative monomers and dimers were summed. L = Ladder; GC = gastric caeca; MT = Malpighian tubules; HG = Hindgut. (TIF) [file pone.0234892.s002.tif]

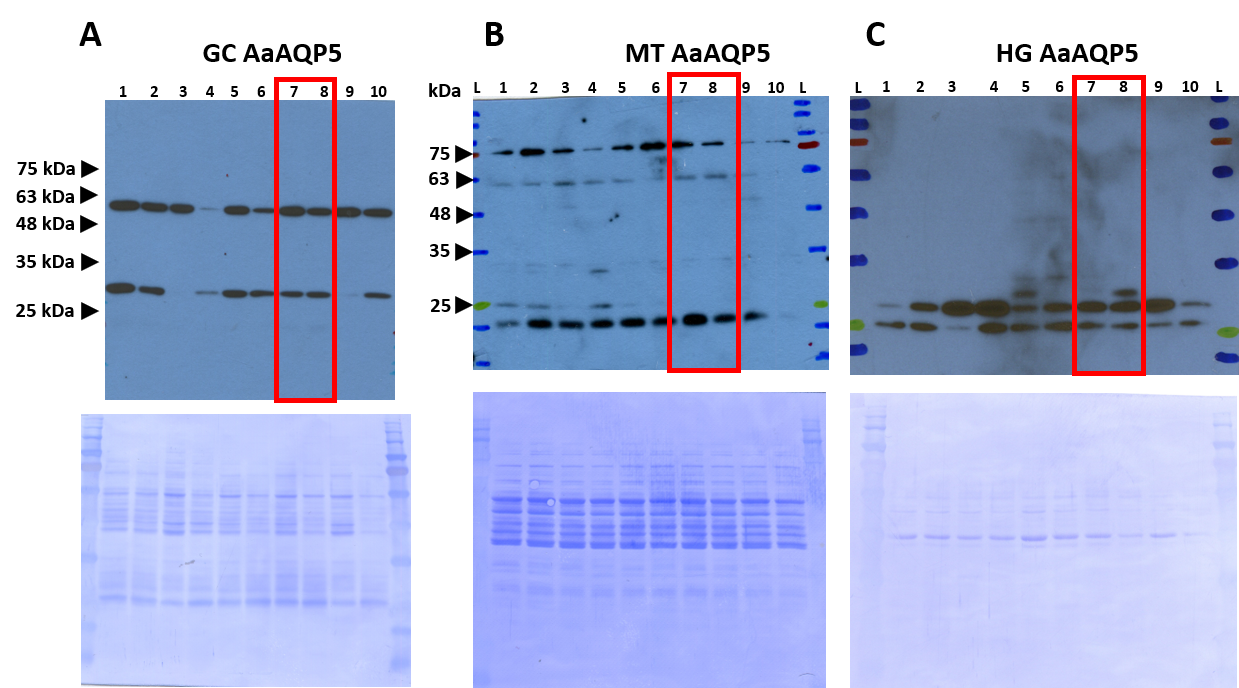

Supplement: S3 Fig — Five biological replicates of organ protein homogenates from freshwater reared larvae are loaded in lanes 1, 3, 5, 7 and 9. Five biological replicates of organ protein homogenates from brackish water reared larvae are loaded in lanes 2, 4, 6, 8 and 10. The red rectangle indicates the lanes shown in Fig 3. Putative monomers and dimers were used for protein quantification. L = Ladder; GC = gastric caeca; MT = Malpighian tubules; HG = Hindgut. (TIF) [file pone.0234892.s003.tif]
