# Supplementary material for: A comparison of aquaporin expression in mosquito larvae (Aedes aegypti) that develop in hypo-osmotic freshwater and iso-osmotic brackish water
Source: PLoS One. 2020 Aug 20;15(8):e0234892. doi: 10.1371/journal.pone.0234892 (PMC7440623; doi:10.1371/journal.pone.0234892)
Supplement: Attachment — Submitted filename: Responses to Reviewer Comments.docx [file pone.0234892.s007.docx]

Reviewer #1: This study examined expression of aquaporins in Aedes larvae reared under brackish and fresh water conditions. The general conclusion is that there is little change in the AQP levels, which suggests that there is little role in AQPs in relation to adapting to brackish waters.

My only major concern is without functional studies, such as RNAi, that that role of AQPs shoudl not be discounted. It might be worth mentioning there could be a role post-translational. The AQPs could have a role and expressional changes in other tissues.

Also, prolonged rearing of the larvae udner the conditions might have missed the changes that occurred earlier in larval development (worth mentioning, but not a major issue).

*Author Response: These are good points. We have already seen drastic changes in anal papillae in our previous studies. Other areas of the GI tract could also play a role (e.g. midgut) as well as the fatbody which requires further investigation. We have modified the conclusion section at the end of the Discussion to address these points.*

Otherwise, I have few other comments.

Specific points:

1. Check references

*Author Response: Thank you. We have examined the reference section and did spot several errors which have now been corrected.*

2. Double check figure references in text.

*Author Response: We have reviewed our reference to figures in the text and could not find any errors.*

3. I would suggest combining Figure 2-4 into a single figure (since most show no significance).

*Author Response: Good suggestion. We had previously combined all these blots into a very large multi-panel figure but we felt this created confusion in terms of which aqp protein the images and graphs were corresponding to. We therefore elected to separate them and feel that this makes it much clearer for readers.*

4. Arrow labeling isn't consistent on Figure 5.

*Author Response: We have carefully reviewed the arrow labels on Figure 5 and how these are explained in the figure caption and how they are explained in the results text of the manuscript. We could not find any errors in these labels and how they are explained.*

5. qPCR: Please provide evidence that 18s RNA (control).

*Author Response: Thank you. The expression values for 18S in all organs of FW and BW reared larvae of this study are provided in the supplementary file for qPCR data. The validity of using 18s RNA was also previously shown by Jonusaite et al 2016. We have now included this reference with the statement about 18s RNA in the Methods section.*

6. Provide df and F values for stats.

*Author Response: We have now included an excel spreadsheet with all of the statistical values for the qPCR and western blot quantitative data. The spreadsheet is provided with supplementary data.*
